# Managing the life cycle of a portfolio of open data resources at the SIB Swiss Institute of Bioinformatics

**DOI:** 10.1093/bib/bbab478

**Published:** 2021-11-30

**Authors:** Chiara Gabella, Severine Duvaud, Christine Durinx

**Affiliations:** Severine DuvaudSIB Swiss Institute of Bioinformatics, Quartier Sorge - Bâtiment Amphipôle, CH-1015 Lausanne, Switzerland; Severine DuvaudSIB Swiss Institute of Bioinformatics, Quartier Sorge - Bâtiment Amphipôle, CH-1015 Lausanne, Switzerland; Severine DuvaudSIB Swiss Institute of Bioinformatics, Quartier Sorge - Bâtiment Amphipôle, CH-1015 Lausanne, Switzerland

**Keywords:** bioinformatics, research infrastructure, User Experience (UX), best-in-class resources, sustainability, open science

## Abstract

Data resources are essential for the long-term preservation of scientific data and the reproducibility of science. The SIB Swiss Institute of Bioinformatics provides the life science community with a portfolio of openly accessible, high-quality databases and software platforms, which vary from expert-curated knowledgebases, such as UniProtKB/Swiss-Prot (part of the UniProt consortium) and STRING, to online platforms such as SWISS-MODEL and SwissDrugDesign. SIB’s mission is to ensure that these resources are available in the long term, as long as their return on investment and their scientific impact are high. To this end, SIB provides its resources, in addition to stable financial support, with a range of high-quality, innovative services that are, to our knowledge, unique in the field. Through this first-class management framework with central services, such as user-centric consulting activities, legal support, open-science guidance, knowledge sharing and training efforts, SIB supports the promotion of excellence in resource development and operation. This review presents the ecosystem of data resources at SIB; the process used for the identification, evaluation and development of resources; and the support activities that SIB provides. A set of indicators has been put in place to select the resources and establish quality standards, reflecting their multifaceted nature and complexity. Through this paper, the reader will discover how SIB’s leading tools and databases are fostered by the institute, leading them to be best-in-class resources able to tackle the burning matters that society faces from disease outbreaks and cancer to biodiversity and open science.

## Introduction

**Figure 1:**
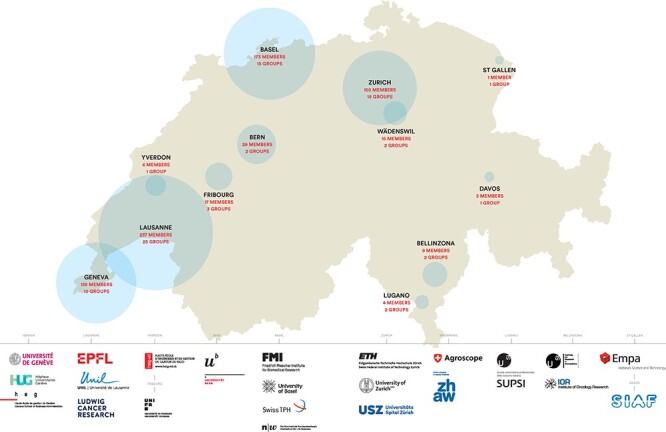
Map of SIB partner institutions as of July 2021.

The SIB Swiss Institute of Bioinformatics (www.sib.swiss) is an internationally recognized non-profit organization, which is dedicated to biological and biomedical data science. It is present in the main academic institutions in Switzerland (Figure 1) and leads numerous national and international projects with a major impact on life science research and health.

SIB’s scientists create knowledge and convert complex questions into solutions in many fields, ranging from biodiversity and evolution to medicine. They provide essential resources, such as databases and software platforms, as well as data management, software engineering, biocuration services, computational biology know-how and training in bioinformatics. The institute delivers this expertise to academic groups and clinicians as well as to private companies.

SIB federates the Swiss bioinformatics community of some 800 scientists, encouraging collaboration and knowledge sharing. It also cooperates with national and international institutions on research infrastructure matters. The institute contributes to keeping Switzerland at the forefront of innovation by promoting progress in biological research and by enhancing health.

Although data resources play an essential role in life science research and are often taken for granted by the scientific community, their sustainability is often very uncertain. A study by Attwood *et al*. examined the 18-year survival of 326 publicly available biological databases and found that >60% of them had ‘died’ during this period, leading to the data no longer being accessible. A further 14% had been archived and were therefore no longer updated [1]. This situation is strongly linked with the fact that most data resources have no guaranteed long-term funding and are dependent on grants that are often shorter in duration than the resource’s typical planning horizon. Resources anchored in institutions are, in general, more likely to survive since they have the benefit of continuous institutional support. Nonetheless, a sustainable funding model that ensures their maintenance and development remains a critical challenge [2]. Recently, funders worldwide have created the Global Biodata Coalition with the mission to stabilize and ensure sustainable financial support for global biodata infrastructure. In particular, the goal is to identify a set of Global Core Biodata Resources (GCBRs) that are crucial for sustaining the broader biodata infrastructure [3] for prioritized long-term support. These GCBRs extend to the entire world the Core Data Resources concept developed by ELIXIR to identify a set of data resources that are fundamental for life science data infrastructure [4, 5] in Europe. SIB is very active in these initiatives with years of commitment to ensuring the financial stability and sustainability of data resources.

Against this background, SIB’s mission is to provide the national and international life science community with a state-of-the-art bioinformatics infrastructure, including resources, expertise and services. Since 2000, the State Secretariat for Education, Research and Innovation (SERI) has been engaged in supporting this international research infrastructure by providing stable funding to SIB for the provision of bioinformatics resources to the life science community. As a result, SIB invests close to CHF 11 million per year in its resources, corresponding to approximately 75 full-time equivalent (FTE) positions, CHF 6.5 million of which comes from SERI. In addition, the Swiss schools of higher education contribute with around 24 FTEs. Within the limits of the available funding, SIB’s commitment is to ensure the long-term existence of SIB Resources and to provide a stable environment for the development and maintenance of high-quality databases and software tools.

This paper gives an overview of the methods used to support the identification, evaluation, development and management of SIB bioinformatics resources. Therefore, it is meant for (i) scientists developing data resources, (ii) universities and funding agencies to improve their selection and evaluation processes and (iii) international initiatives to enable the sustainability of data resources. It also describes the network of data resources at SIB, their dependencies and how they interact and form a solid foundation for the life science community.

## Resources at SIB

**Figure 2:**
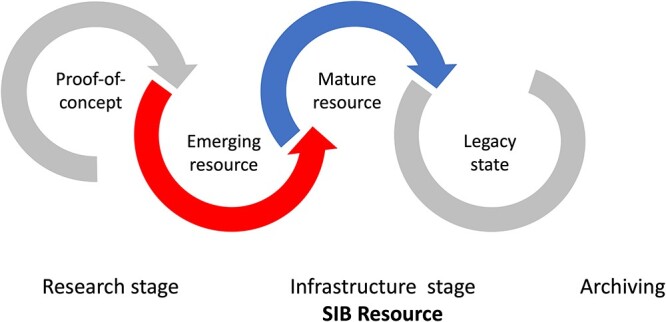
The life cycle of a bioinformatics resource.

A new ‘resource’, i.e. database, service or software tool, typically results from a research project that leads to a proof of concept. With further development, it can evolve toward maturity and, if successful, may become part of the research infrastructure available to the scientific community. If the resource is no longer relevant to the scientific community, the decision may be taken to archive it. This stage is referred to as ‘Legacy’ in [4]. It is generally accepted that the resource is permanently archived after 1 year in the ‘Legacy’ phase (Figure 2).

The scientific groups, that are part of SIB, develop and maintain >160 resources that are made available to the global scientific community through Expasy, the Swiss bioinformatics resource portal (www.expasy.org). The portal offers comprehensive and up-to-date information on the resources through a collaborative effort and careful annotation by SIB Resource Providers. The resources are described using a standardized ontology, connecting functionally related resources and allowing the exploration of the network of resources [6].

**Figure 3:**
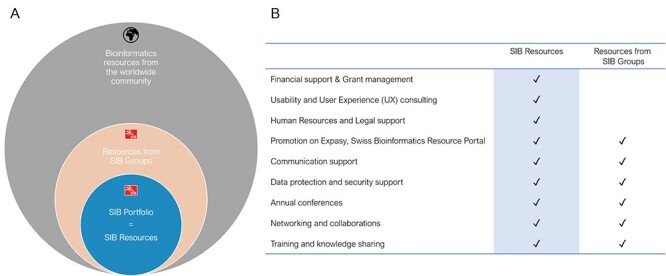
SIB Resources and resources from SIB groups. How they are embedded in the global bioinformatics community (**A**) and the services that SIB offers to them (**B**).

Among this rich ecosystem of high-quality resources, a subset of resources is carefully selected to be part of the SIB portfolio. These are referred to as SIB Resources (Figure 3**A**)*.* They represent a collection of openly available resources of particular importance to the life science community. These are defined as best in class based on three criteria:

(i) Scientific impact: Does the resource show high levels of usage within its target audience? Does it fill an important unmet need of the scientific community? Does it provide excellent scientific quality and service? Is it considered an authority in its field?
(ii) Scientific return on investment: Is the impact of the resource on the life science community satisfactory in relation to the financial investment? Is the difference it makes for the community worth the investment? Is the resource best in class compared to its competitors?
(iii) Fit within the SIB Resource portfolio and strategic orientation.

These resources benefit from the institute’s specific funding support and services in addition to the other multiple advantages offered by the SIB to its community (Figure 3**B** and see the ‘SIB services and support for SIB Resources’ section). Far from being static, this portfolio includes everything from emerging to well-established resources and is regularly evolving in response to scientific progress and changes in researchers’ needs. The selection process and indicators used are described in detail in the ‘SIB Resources: selection and evaluation process’ section, and the full list of SIB Resources in 2021 is available in Table 1.

## Examples of SIB resources

### Databases

The most widely known SIB Resource is the UniProtKB/ Swiss-Prot database, which is part of the UniProt Consortium [7, 8]. The knowledgebase contains a reviewed collection of high-quality annotated and non-redundant protein sequences, bringing together experimental results, computed features and scientific conclusions to provide information related to a protein’s function, structure and subcellular location, specific features and interactions. The UniProtKB/Swiss-Prot database contains >500 000 protein sequences curated by experts with the support of advanced machine learning techniques [9]. With nearly 2 million unique users per month, UniProt is the most widely used protein information resource in the world.

STRING is a knowledgebase and software tool for known and predicted protein–protein interactions [10]. It includes direct (physical) and indirect (functional) associations derived from various sources, such as genomic context, high-throughput experiments, (conserved) co-expression and the literature. STRING networks cover over 5000 different organisms with >25 million high-confidence links between proteins.

### Services and software tools

The SWISS-MODEL Workspace [11] is a fully automated web-based service, which assists and guides the user in building a three-dimensional structure of a protein based on its homology with proteins for which experimentally determined structures are available. SWISS-MODEL receives over a million model requests every year.

SwissDrugDesign is a comprehensive suite of web-based computer-aided drug design tools, varying from molecular docking to pharmacokinetics and druglikeness, among others. SwissDrugDesign has been used massively during the recent COVID-19 pandemic, with >1.5 million jobs submitted during 2020 alone.

**Table 1:** SIB Resources as of 2021

Name of SIB Resource		Type	Description	Highlights
ASAP	Portal for single-cell data analysis	Software tool	Web-based, collaborative portal aimed at democratizing single-cell omics data analyses. Provides a full modular single-cell ribonucleic acid (RNA)-seq analysis pipeline	Enables standardized analyses that can be run in minutes by any user without requiring significant computing power. Joined the SIB portfolio in 2021
Bgee	Gene expression expertise	Knowledgebase with expert curation and software tools	Gene expression data (including all types of transcriptomes), allowing retrieval and comparison of expression patterns between animals, humans, model organisms and diverse species of evolutionary or agronomical relevance	Only resource to provide homologous gene expression between species
Cellosaurus	Knowledge resource on cell lines	Knowledgebase with expert curation and software tools	Information on all cell lines used in biomedical research, including immortalized cell lines, naturally immortal cell lines (stem cell lines), finite life cell lines, vertebrate cell lines with an emphasis on human, mouse and rat cell lines and invertebrate (insect and tick) cell lines	An ELIXIR Core Data Resource. Cellosaurus contains 128 000 entries with about 50 different types of information items, ~21 000 literature references and cross-references to 93 resources. Joined the SIB portfolio in 2021
EPD	Eukaryotic Promoter Database	Knowledgebase with expert curation and software tools	Quality-controlled information on experimentally defined promoters of higher organisms, as well as web-based tools for promoter analysis.	Over 180,000 downloadable promoters that can be analyzed over a web interface and viewed in the UCSC genome browser.
Glyco@Expasy	Zooming in on web-based glycoinformatics resources	Knowledgebase with expert curation and software tools	Centralized web-based glycoinformatics resources developed within an international network of glycoscientists. Aims of (i) popularizing the use of bioinformatics in glycobiology and (ii) emphasizing the relationship between glycobiology and protein-oriented bioinformatics resources	Completely redesigned with glycoscientists in 2021 to meet the growing needs of the community. Joined the SIB portfolio in 2021
neXtProt	Human protein knowledgebase	Knowledgebase with expert curation and associated tools	Information on human proteins, such as function, involvement in diseases, messenger ribonucleic acid/protein expression, protein/protein interactions, post-translational modifications, protein variations and their phenotypic effects	High data coverage through integration of multiple sources. Advanced semantic search functionalities. Tools specifically designed for the proteomics community
Nextstrain	Impact of pathogen genome data on science and public health	Software tool	Open-source project to harness the scientific and public health potential of pathogen genome data. Provides a continually updated view of publicly available data alongside powerful analytic and visualization tools for use by the community	Started as an influenza-specific project, then evolved to Ebola, Mers, Zika and SARS-CoV-2. Joined the SIB portfolio in 2021
Rhea	Expert-curated database on biochemical reactions	Knowledgebase with expert curation	Knowledgebase of chemical and transport reactions of biological interest and the standard for enzyme and transporter annotation in UniProtKB	An ELIXIR Core Data Resource. Rhea contains >13 000 reactions with ~12,000 unique compounds
STRING	Protein–protein interaction networks and functional enrichment analysis	Knowledgebase and software tool	Resource for known and predicted protein–protein interactions, including direct (physical) and indirect (functional) associations derived from various sources, such as genomic context, high-throughput experiments, (conserved) co-expression and the literature	An ELIXIR Core Data Resource. STRING networks cover >5000 different organisms, with >25 million high-confidence links between proteins
SwissDrugDesign	Widening access to computer-aided drug design	Software tools	Web-based computer-aided drug design tools ranging from molecular docking (SwissDock) to pharmacokinetics and druglikeness (SwissADME), through virtual screening (SwissSimilarity), lead optimization (SwissBioisostere) and target prediction of small molecules (SwissTargetPrediction)	Comprehensive and integrated web-based drug design environment
SWISS-MODEL	Protein structure homology-modeling	Software tool and repository	Automated protein structure homology-modeling platform for generating 3D models of a protein using a comparative approach and database of annotated models for key reference proteomes based on UniProtKB	Easy-to-use web-based platform processing >2 million model requests per year, providing model information for experts and non-specialists
SwissOrthology (OMA + OrthoDB)	One-stop shop for orthologs	Phylogenomic databases and software tools	Web portal of resources to infer orthologs, i.e. corresponding genes across different species, a key aspect to predicting gene function or reconstructing species trees. It includes OrthoDB, BUSCO as well as OMA and the Quest for Orthologs benchmark service	World-leading orthology and comparative genomic resources
SwissRegulon	Tools and data for regulatory genomics	Software tools and knowledgebases	Web portal for regulatory genomics, including genome-wide annotations of regulatory sites and motifs, the webserver ISMARA for automated inference of regulatory networks and CRUNCH for automated analysis of ChIP-seq data and REALPHY for reconstructing phylogenies from raw sequence data	ISMARA and Crunch web servers allow users to upload raw microarray, RNA-seq or ChIP-seq data to automatically infer the core regulatory networks acting in their system of interest
UniProtKB/SwissProt	Protein knowledgebase	Knowledgebase with expert curation	Hundreds of thousands of protein descriptions, including function, domain structure, subcellular location, post-translational modifications and functionally characterized variants	Expert-curated part of UniProt, the most widely used protein information resource in the world, with >6 million pageviews per month. An ELIXIR Core Data Resource
SwissLipids	A knowledge resource for lipids	Knowledgebase with expert curation	Information about known lipids, including knowledge of lipid structures, metabolism and interactions, providing a framework for the integration of lipid and lipidomics data with biological knowledge and models	Contains information on >590 000 lipid structures from >640 lipid classes
V-pipe	Viral genomics pipeline	Software tool	Pipeline integrating various open-source software packages for assessing viral genetic diversity from NGS data	Enabling reliable and comparable viral genomics and epidemiological studies and facilitating clinical diagnostics of viruses

### Emerging resources

The SIB Resource portfolio also contains more recent resources, such as SwissLipids (a knowledgebase of lipid structures, metabolic reactions, enzymes and interacting proteins [12]) or V-pipe [a pipeline for assessing viral genetic diversity from next-generation sequencing (NGS) data] [13]. Like SwissDrugDesign, V-pipe has played a crucial role during the COVID-19 pandemic. The tool, redeployed for SARS-CoV-2, is used to analyze most of the Swiss samples and to identify the variants circulating in the country. This response to the recent crisis involved developing new features in existing software tools or repurposing them and issuing new releases in record time through coordinated national and international efforts. It was made possible by SIB’s scientists’ dedication and by the fact that most resources were available with sustainable funds and with teams ready to operate the necessary developments. Indeed, long-term funding gives scientists the opportunity to temporarily readapt their main mission and allows for flexibility and resilience in responding to a crisis.

### Measuring the impact and excellence of SIB Resources

SIB Resources are evaluated and monitored through a set of 27 indicators grouped into 6 categories (Table 2). Indicators are assessed differently depending on the type of resource (databases or software tools): the whole body of indicators together reflects the quality and impact of a specific bioinformatics resource. The term ‘indicators’ is used rather than ‘metrics’ to evaluate resources: indeed, while a ‘metric’ suggests that the impact of bioinformatics resources could be easily ‘measured’, an ‘indicator’ is defined as a measurable quantity that substitutes for something that is less easily measured. For example, the number of citations could be used as an indicator of scientific impact even though scientific impact may exist in a way that does not generate citations. Indicators must therefore be interpreted using expertise, insight and caution [4].

**Table 2:** The six categories covering the 27 quantitative and qualitative indicators (more on [16])

	Category	Description	Number of indicators included
I	Scientific focus and quality of science	Demonstrate high quality of data and metadata, respond to a clear scientific need and be unique. This implies benchmarking against other resources and being an authority in its field compared to the major competitors	Four
II	Community	Know the community to whom it is addressed, its size and usage: web statistics, user reach and community size. Candidate Resources with a valid track record of usage, responding to a clear need within the scientific community are more likely to be included as SIB Resources. However, emerging resources are encouraged to submit as well. The scientific context in which the resource operates should be taken into account. A resource that serves a small scientific community may not have as many users as a resource serving a broader interest, and yet, it may reach 90% of the community it supports (coverage) and be crucial for the scientific work of that community	Five
III	Quality of service	Demonstrate a high level of service and reliability with the integration of features, such as persistent and unique identifiers, community-recognized standards, user support and training and integration of user feedback	Five
IV	Legal and funding infrastructure and governance	Have a sound legal framework supporting open science and seek complementary funds from other sources in order to ensure sustainable long-term funding	Four
V	Impact and translational stories	Have a significant impact on the life science community and be impact-driven	Three
VI	SIB	Contribute to SIB in terms of scientific credibility and visibility and demonstrate alignment and synergies with the current portfolio of SIB Resources	Three

**Figure 4:**
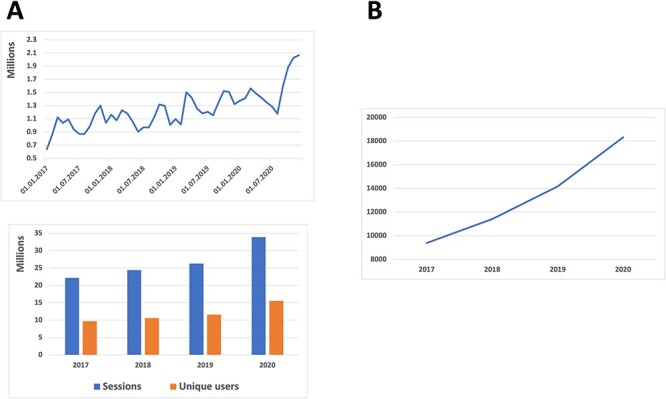
Change in the usage and impact of the data resources in the SIB portfolio. (**A**) Bottom: the bar chart represents the aggregated values of unique users per year (orange bars) and sessions per year (blue bars) across all SIB Resources (data from Google Analytics). Top: the blue line indicates the upward trend of cumulative unique users per month accessing SIB Resources. (**B**) Impact and usage in research of SIB data resources as measured through aggregated citations in the literature (source: Europe PMC). The datasets used to generate the graphs are available in GitHub: https://github.com/sib-swiss/managing-life-cycle-portfolio-sib-resources/blob/main/Resources-statistics.xlsx.

The use of bioinformatics resources has become increasingly important in academia and industry in recent years. Nowadays, they are essential for ensuring the reproducibility and integrity of research [14]. SIB Resources are no exception. Figure 4 illustrates the change in the use and impact of the SIB Resource portfolio over the past 4 years, demonstrating the growing importance of these resources in life sciences. As an indicator of the level of usage of SIB Resources, information about visits and visitors is collected using Google Analytics. As this web analytics service does not consider other types of user interaction, such as FTP or programmatic access, and as its tracking tags may be blocked by adblockers, it underestimates the overall usage. Citation indicators (Figure 4**B**) are a means of showing the usage of SIB Resources in research projects and are therefore also part of the impact indicators. The full text of open-access publications in Europe PMC can be searched with the SIB Resource names. As with any indicator, the numbers must be interpreted with care: citations obtained from Europe PMC are limited to open-access literature and are therefore also an underestimate of usage in research. They can be used, however, to observe trends. Furthermore, the more widespread the use of a resource, the more it becomes a common feature of research practice and is no longer cited [5, 15]. Given these caveats, the upward trends show the ever-increasing impact of the SIB Resources in research.

Since databases and software tools are very diverse, it is crucial to consider their many different facets when selecting indicators for evaluation. These indicators are reviewed regularly to adapt to the latest changes in science and to support the promotion of excellence aligned with international standards. Providing precise information and figures informs reviewers and experts and allows them to make objective recommendations. These indicators can also be helpful for the scientists developing a resource to guide the development process.

### Prerequisites for the selection of SIB Resources

The SIB portfolio brings together resources that are beyond the proof-of-concept stage and have reached a sufficient level of maturity to be considered as infrastructure. Candidate Resources must prove to be unique in the open-science landscape and fit within the SIB Resource portfolio in order to enable synergies with the other resources.

The resources need to fill a specific need of the research community and demonstrate high scientific quality, impact and visibility. The resources must comply with international standards. Resource Providers need to demonstrate a solid knowledge of their target user community, both qualitatively and quantitatively. They should make continuous efforts to ensure that their resource meets user needs and expectations by providing a helpdesk and should include user input in their development and implementation plans.

The adoption of open science practices is also a key component in resource selection. Indeed, SIB’s vision is that data and research results should be freely accessible to all to increase scientific collaboration and data transparency. In this respect, SIB is committed to open access as a core principle for public research data. For this reason, the institute promotes the adoption of open licenses for resources, results and data generated through public funds, such as Creative Commons and GNU licenses, unless a different licensing model is more appropriate for specific cases. Most resources at SIB are therefore both technically open (data are available in a machine-readable standard format, which means they can be retrieved and meaningfully processed by a computer application) and legally open (explicitly licensed in a way that permits commercial and non-commercial use and re-use without restrictions).

### SIB Resources as a network

**Figure 5:**
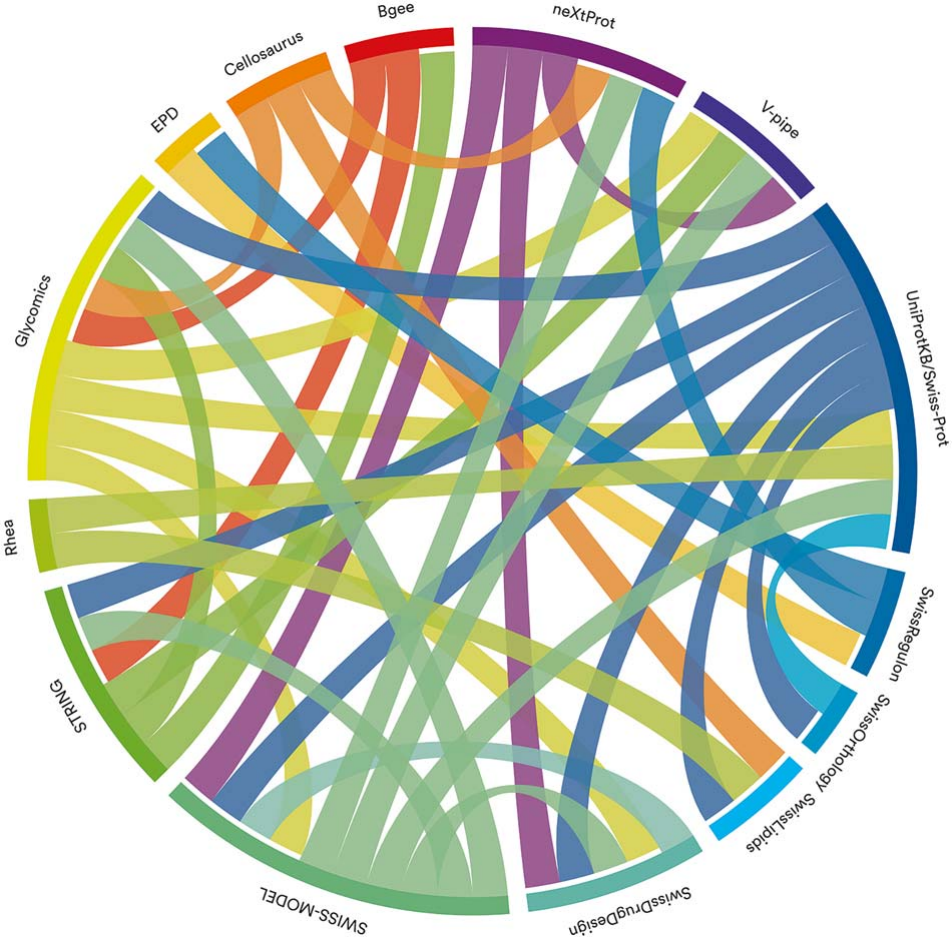
Data resources in the SIB ecosystem are highly interconnected: chord diagram showing the data flows between the resources (the flow has the same color as the resource of origin). The image was produced using Circos circos.ca. The datasets used to generate the diagram are available in GitHub: https://github.com/sib-swiss/managing-life-cycle-portfolio-sib-resources/blob/main/matrices-data-flow.xlsx.

SIB Resources are highly interconnected. They are part of an ecosystem with strong dependencies, which forms a solid foundation on which scientists can rely. Indeed, some resources may be the source of data for other resources (Figure 5 shows the data flow across resources). There are also many cross-links between resources. These allow users to navigate from one resource to another, thus extending the scope of exploration. SIB Resources are encouraged to strengthen this interconnectivity.

### SIB Resources: selection and evaluation process

SIB has opted for a performance-driven selection and evaluation process. This ensures that the SIB Resources are the best in class, state of the art and aligned with the needs and expectations of life scientists worldwide.

Every 4 years, the SIB Resource Providers (i.e. the groups that develop resources that are already part of the SIB portfolio) submit a workplan for the forthcoming funding period, including objectives and an implementation plan. The workplans submitted by the Resource Providers are based on the set of 27 indicators mentioned earlier (Table 2; a template of the workplan with a list of indicators is available in [16]). In addition, they must describe their latest achievements, together with the status of the recommendations made in the previous evaluation(s). If needed, a review by external experts can be requested to support the further evaluation process.

In parallel, all SIB groups can propose new resources (the so-called Candidate Resources) for inclusion in the SIB portfolio. They submit a workplan containing their objectives and an implementation plan for the next 4 years. These workplans are reviewed and rated by external reviewers.

The SIB Scientific Advisory Board (SAB), a panel of international experts (see https://www.sib.swiss/about-sib/organization#scientific-advisory-board), examines the workplans, reviews the external evaluations and assesses whether the resources meet the best-in-class criteria (i.e. scientific impact, scientific return on investment and fit with the resource portfolio and SIB’s strategic orientation). The most promising Candidate Resources are shortlisted and invited, together with the existing SIB Resources, to the SAB meeting, where the SAB members and the Resource Providers discuss the workplans in more detail.

The SAB provides an overall ranking of the resources as well as recommendations on their inclusion in the resource portfolio and level of funding. This report is used as the basis for the SIB Board of Directors (BoD) to decide on the funding allocation for the upcoming funding period. In addition, this report provides useful insights to the SIB Resource Providers for furthering their development strategy.

**Figure 6:**
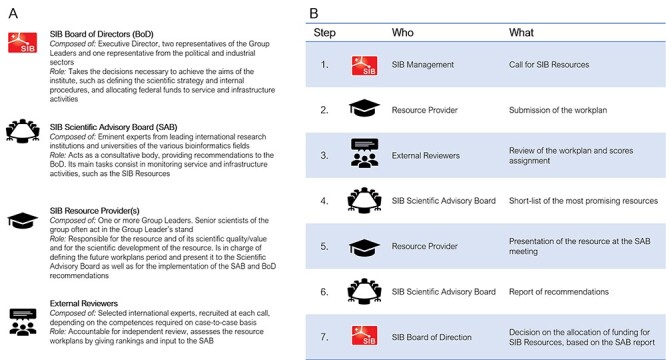
The SIB Resource selection process. Stakeholders (**A**) and procedure (**B**).

Figure 6 describes the main actors in the procedure and represents schematically the various steps of the process in chronological order.

SIB commits to supporting resources as long as their impact across the life science community is high. To this end, 2 years after the beginning of the funding period, SIB Resources are subject to a mid-term review by the SAB. These reviews follow a similar process and use the same indicators as the identification process. In addition, they include an evaluation of the progress made as well as the follow-up on the latest recommendations from the SAB. Based on the outcome, the BoD can decide to adjust the funding level or stop funding the SIB Resource.

### SIB services and support for SIB resources

SIB’s support for SIB Resources takes two forms. Firstly, SIB provides funds for hiring skilled personnel to develop and maintain the resource. Second, SIB Resources have access to a range of high-quality services that support professional infrastructure provision, including User Experience studies and design, hosting, best practice and knowledge sharing, networking opportunities, annual conferences, communication support, assistance from the SIB Data Protection and Security Board, financial services (grant management), human resource support, legal support, training in soft skills and many others (Figure 3**B**). This range of services, which is quite unique in the academic environment, enables further strengthening of the quality of the resource and thus creates a virtuous circle by pushing it to be best in class. The resource is also more likely to receive additional funding from other sources and thus be sustainable in the long term.

The Resource Usability & Support team was created in 2018 to provide a range of high-quality services to SIB Resources to assist them in becoming or remaining the best in class. Like SIB Resources, the group undergoes a 2-yearly assessment by the SAB: this ensures the consistency of activities and the quality of the work provided to the resources.

**Figure 7:**
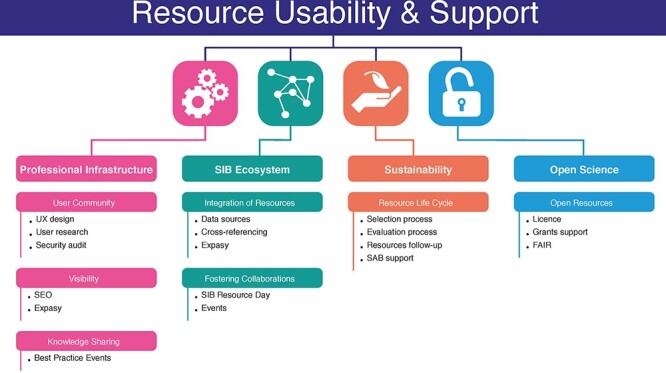
The services offered to assist SIB Resources in becoming and remaining best in class.

The range of services is based on four main pillars (Figure 7): (i) support with respect to professional infrastructure provision, including user research, interviews and workshops, wireframing, etc.; (ii) strengthening of relationships and collaborations between SIB Resources through an annual discussion, networking events and management of Expasy, the Swiss Bioinformatics Resource Portal; (iii) sustainability through managing the SIB Resource selection and evaluation process, including the definition of indicators, coordination and communication with stakeholders—SAB members, the BoD, Resource Providers and external reviewers as well as monitoring and (iv) the promotion of open science.

This panel of activities aims to improve the visibility and quality of SIB Resources so that their impact on the scientific community remains at the highest level.

Indeed, in addition to an established reputation for scientific excellence, usability and visibility play a crucial role in the success of a resource. To conduct their daily work, life scientists and clinicians must navigate an ever-denser forest of tools, software and databases. At the same time, the next generation of researchers—who are both tech-savvy and ardent app-consumers—is raising the bar of expectations in terms of resource usability: scientific excellence is no longer the sole criteria for a resource to be competitive in the long term. For this reason, the team works hand-in-hand with the SIB Resource Providers on the usability of the user interface, the target population of the resources and the usage figures. The principle is that any new resource should be developed first and foremost by consulting its users. A best-practice toolkit to help the resources know and grow their user base along with regular meetings to share their know-how within the community has also been introduced to improve awareness among the resources. Lastly, the establishment of a dedicated User Advisory Board for each SIB Resource, in addition to the SIB SAB, is for SIB among the best practices that are promoted. Such boards are made up of users from academia and industry, power users and/or simple users who conduct scientific and/or technological reviews, ensuring quality and providing *ad hoc* insights and advice to resource managers.

## CONCLUSION

Thanks to its coherent and dynamic portfolio, including both emerging and well-established resources, SIB is a key driver of innovation in bioinformatics. The indicators developed for the evaluation and selection process, through continuous monitoring of usage trends and scientific impact of the resources in the SIB ecosystem, inform their life cycle management by providing strategic recommendations and by allowing them to develop to their full potential. Through the integration of data resources and the establishment of a professional infrastructure, SIB is a cornerstone of excellence in the development, application and management of data resources. Indeed, the provision of a solid professional infrastructure ranging from user-centric design to user research, various types of consulting and funding, enables the SIB’s resource portfolio to be at the forefront of scientific excellence and ensures its long-term sustainability in a context of open science.

**Key Points**

- The SIB Swiss Institute of Bioinformatics provides the life science community with a portfolio of openly accessible, high-quality databases and software platforms.
- Its mission is to ensure that these resources are available and sustainable in the long term.
- A performance-driven selection process ensures that SIB Resources are the best in class and state of the art.
- A high-quality management framework with central services, such as user-centric design, license advice and training efforts, enables the promotion of excellence in resource development and operation.

## Notes

**Chiara Gabella** manages the identification process for SIB Resources and is responsible for their continuous monitoring and the strategy and processes implemented to improve the User Experience (UX). Chiara joined SIB in 2016, after a postdoc in Biophysics (cell motility). She holds an MSc in Mathematical Engineering from the Politecnico in Torino, Italy, and a PhD in Physics from the EPFL, Switzerland.

**Severine Duvaud** is responsible for supporting SIB Resources to increase visibility and to improve the UX. She is also involved in best practices, knowledge and expertise sharing within SIB to reinforce the impact of SIB Resources. Severine joined SIB in 2003. She holds an MSc in Computer Sciences from the National Institute of Applied Sciences in Lyon, France, and an MSc in Biochemistry from the University of Lyon.

**Christine Durinx** is an executive director of the SIB Swiss Institute of Bioinformatics. She joined this non-profit, academic foundation in 2014 as an associate director and was appointed as an executive director in June 2018. Christine holds a degree in Pharmacy and a PhD in Pharmaceutical Sciences. Before joining SIB, she worked in the pharmaceutical industry for >10 years. Christine Durinx was a co-leader of the ELIXIR data platform from 2015 to 2019.
